# The Daidzein Metabolite, 6,7,4'-Trihydroxyisoflavone, Is a Novel Inhibitor of PKCα in Suppressing Solar UV-Induced Matrix Metalloproteinase 1

**DOI:** 10.3390/ijms151121419

**Published:** 2014-11-19

**Authors:** Tae-Gyu Lim, Jong-Eun Kim, Sung-Young Lee, Jun Seong Park, Myung Hun Yeom, Hanyong Chen, Ann M. Bode, Zigang Dong, Ki Won Lee

**Affiliations:** 1 World Class University Biomodulation Major, Department of Agricultural Biotechnology and Center for Food and Bioconvergence, Seoul National University, Seoul 151-742, Korea; E-Mails: tglim83@gmail.com (T.-G.L.); idonlike@gmail.com (J.-E.K.); 2 The Hormel Institute, University of Minnesota, Austin, MN 55912, USA; E-Mails: slee@hi.umn.edu (S.-Y.L.); hchen@hi.umn.edu (H.C.); bodex008@umn.edu (A.M.B.); 3 Advanced Institutes of Convergence Technology, Seoul National University, Suwon 443-270, Korea; 4 Skin Research Institute, Amorepacific Corporation R&D Center, Yongin 446-829, Korea; E-Mails: superbody@amorepacific.com (J.S.P.); mhyeom@amorepacific.com (M.H.Y.); 5 Research Institute of Bio Food Industry, Institute of Green Bio Science and Technology, Seoul National University, Pyeongchang 232-916, Korea

**Keywords:** matrix metalloproteinase 1, protein kinase C (PKC)α, photoaging, 6,7,4'-trihydroxyisoflavone

## Abstract

Soy isoflavone is an attractive source of functional cosmetic materials with anti-wrinkle, whitening and skin hydration effects. After consumption, the majority of soy isoflavones are converted to their metabolites in the human gastrointestinal tract. To understand the physiological impact of soy isoflavone on the human body, it is necessary to evaluate and address the biological function of its metabolites. In this study, we investigated the effect of 6,7,4'-trihydroxyisoflavone (6,7,4'-THIF), a major metabolite of daidzein, against solar UV (sUV)-induced matrix metalloproteinases (MMPs) in normal human dermal fibroblasts. MMPs play a critical role in the degradation of collagen in skin, thereby accelerating the aging process of skin. The mitogen-activated protein/extracellular signal-regulated kinase (MEK)/extracellular signal-regulated kinase (ERK), mitogen-activated protein kinase (MKK)3/6/p38 and MKK4/c-Jun *N*-terminal kinases (JNK) signaling pathways are known to modulate MMP-1 function, and their activation by sUV was significantly reduced by 6,7,4'-THIF pretreatment. Our results also indicated that the enzyme activity of protein kinase C (PKC)α, an upstream regulator of MKKs signaling, is suppressed by 6,7,4'-THIF using the* in vitro* kinase assay. Furthermore, the direct interaction between 6,7,4'-THIF and endogenous PKCα was confirmed using the pull-down assay. Not only sUV-induced MMP-1 expression, but also sUV-induced signaling pathway activation were decreased in PKCα knockdown cells. Overall, we elucidated the inhibitory effect of 6,7,4'-THIF on sUV-induced MMPs and suggest PKCα as its direct molecular target.

## 1. Introduction

With the continued increase of the elderly population in modern society, the demand for ways to look younger is also rising. The prevention of skin aging is vital for maintaining a younger-looking appearance [1]. Accordingly, preventing or reversing skin aging has become a central subject of research in the dermatological and cosmeceutical fields [2]. Skin aging is a naturally occurring process of senescence and is accompanied by clinical signs, such as sagging, fine wrinkles and paleness [3,4]. Genetic and environmental factors contribute to the aging of skin. In particular, exposure to sunlight is a key factor contributing to the acceleration of the skin aging process [5]. Skin that is excessively exposed to sunlight shows a decline of skin functions and features deep wrinkles, dryness and uneven pigmentation [4,6,7], which is a process termed photoaging [1,8,9]. Ultraviolet (UV) light is mainly responsible for the skin damaging effect of sunlight. Recent research is focusing on developing strategies to prevent UV-induced biochemical changes in the skin, thereby alleviating skin photoaging.

Human skin tissue is composed of various extracellular matrix (ECM) components, such as collagen, keratin and elastin. Skin wrinkles are primarily formed as a result of collagen breakdown. While various enzymes participate in the degradation of ECM, matrix metalloproteinases (MMPs) are the key enzymes for degrading ECM with substrate specificity [10,11,12]. Among them, MMP-1 is the primary enzyme that degrades collagen in human skin [13,14]. Although various environmental factors can activate MMP-1 expression, solar UV (sUV) is a major inducer of MMP-1 expression in skin [12]. Thus, suppression of sUV-induced MMP-1 expression is regarded as a powerful strategy for development of anti-photoaging agents.

**Figure 1:**
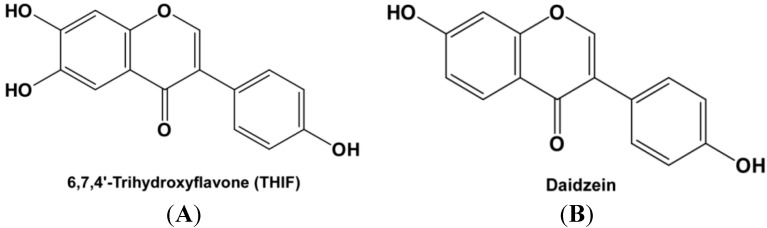
Molecular structure of 6,7,4'-trihydroxyisoflavone (6,7,4'-THIF) (**A**) and daidzein (**B**).

Multiple lines of evidence have reported the health benefits of soy isoflavones [15,16,17,18]. In particular, daidzein has been shown to have a beneficial effect on skin health [19] and has a high amount of daily intake [20]. 6,7,4'-trihydroxyisoflavone (6,7,4'-THIF, Figure 1A) is one of the major derivatives converted from daidzein [21]. We previously reported the pharmacological activities of daidzein derivatives [15,16,18], including the anti-cancer effects of 7,3',4'-THIF and 6,7,4'-THIF in skin and colon. The anti-atopic dermatitis effect of 7,3',4'-THIF was reported in the NC/Nga Mice model [22]. However, the effect of 6,7,4'-THIF and its molecular target in skin aging have not been elucidated yet.

In the present study, we aimed to investigate the anti-skin photoaging effect of 6,7,4'-THIF. We showed that 6,7,4'-THIF attenuated sUV-induced MMP-1 expression more effectively than its precursor, daidzein, by directly suppressing protein kinase C (PKC)α kinase activity, suggesting its potential protective effect against skin photoaging.

## 2. Results

### 2.1. 6,7,4'-Trihydroxyisoflavone (6,7,4'-THIF) Reduces Solar UV (sUV)-Induced Matrix Metalloproteinase (MMP)-1 Expression in Normal Human Dermal Fibroblasts (NHDF) Cells

**Figure 2:**
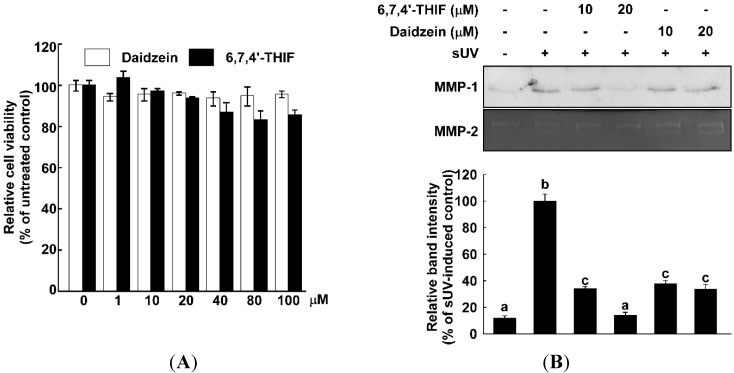
6,7,4'-THIF suppresses solar UV (sUV)-induced MMP-1 expression in normal human dermal fibroblasts (NHDF) cells with a non-cytotoxic concentration range. (**A**) To determine the non-cytotoxic concentration range of the compounds, [3-(4,5-dimethylthiazol-2-yi)-5-(3-carboxymethoxyphenyl)-2-(4-sulfophenyl)-2H-tetrazolium, inner salt] (MTS) analysis was performed. The cells were cultured to confluence in 96-well plates, and then, the cells were treated for 24 h with daidzein and 6,7,4'-THIF (1, 10, 20, 40, 80 and 100 µM). The cell viability was determined using MTS analysis, as described in the Materials and Methods; and (**B**) 6,7,4'-THIF shows a better inhibitory effect than its precursor, daidzein, on sUV-induced MMP-1 expression. After 1 h of treatment with each compound, the cells were exposed to sUV. The secreted MMP-1 was measured using western blot analysis, as described in the Materials and Methods. Data are representative of three independent experiments, which gave similar results. Means with different letters (a–c) within a graph are significantly different from each other at *p* < 0.05.

Accumulative data have shown that UV radiation induces MMP-1 expression, which is a key responsible mechanism in UV-induced skin wrinkle formation [12,13,23,24]. Hence, the inhibition of MMP-1 expression serves as an excellent strategy in developing anti-wrinkle agents. We compared the inhibitory effect of daidzein and its metabolite, 6,7,4'-THIF, on sUV-induced MMP-1 expression in normal human dermal fibroblasts (NHDF) cells. MMP-1 expression was markedly elevated by sUV irradiation, and 6,7,4'-THIF suppressed sUV-induced MMP-1 expression in a dose-dependent manner (Figure 2A). On the other hand, daidzein did not lead to a significant reduction of sUV-induced MMP-1 expression at the concentration used in this study (10 or 20 μM) in NHDF cells. Daidzein and 6,7,4'-THIF did not exhibit cytotoxicity at the concentration we used in this study (Figure 2B). Taken together, 6,7,4'-THIF shows a better inhibitory effect on sUV-induced MMP-1 expression than its precursor, daidzein.

### 2.2. 6,7,4'-THIF Inhibits sUV-Induced Mitogen-Activated Protein Kinase(MAPK) Signaling Pathways in NHDF Cells

**Figure 3:**
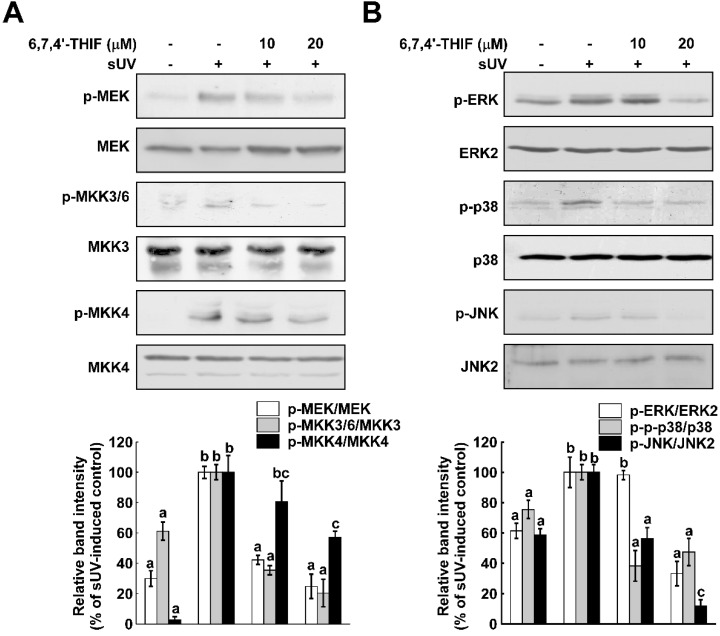
6,7,4'-THIF down-regulates sUV-induced MAPKK and MAPK activation. To determine the effect 6,7,4'-THIF on the sUV-induced MAPKK (**A**) and MAPK (**B**) signaling pathway, the phosphorylation level of the signaling proteins was analyzed by western blot, as described in the Materials and Methods. After pretreatment with 6,7,4'-THIF (10 and 20 µM) for 1 h, the cells were exposed to sUV. The cell lysates were collected after an additional 15 min (p-MEK, p-MKK3/6 and p-MKK4) and 30 min (p-ERK, p-p38 and p-JNK). Data are representative of three independent experiments, which gave similar results. Means with different letters (a–c) within a graph are significantly different from each other at *p* < 0.05.

MMP-1 is regulated by various inflammatory signaling pathways, including the MAPK pathways [25,26]. To determine if MAPKs are involved in the inhibitory function of 6,7,4'-THIF on sUV-induced MMP-1 expression, we evaluated the effect of 6,7,4'-THIF on sUV-induced MAPK phosphorylation in NHDF cells. Similar to previous studies, phosphorylation of MAPKKs and MAPKs was induced by sUV exposure, and 6,7,4'-THIF reduced sUV-induced upregulation of the MEK-ERK, MKK4-JNK and MKK3/6-p38 signaling pathways (Figure 3). As all of the MAPKK phosphorylation was decreased by 6,7,4'-THIF in NHDF cells, we hypothesized that 6,7,4'-THIF may regulate an upstream modulator of MAPKKs.

### 2.3. 6,7,4'-THIF Suppresses Protein Kinase C (PKC)α Kinase Activity with Direct Binding

**Figure 4:**
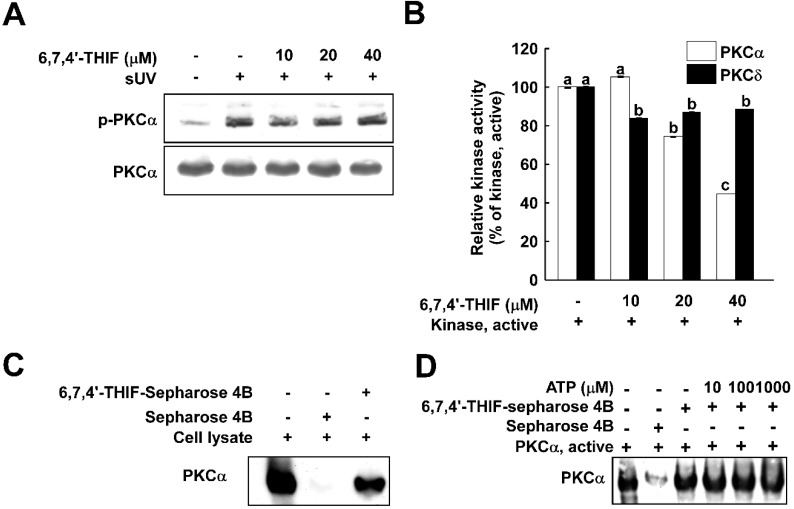
PKCα is a direct target of 6,7,4'-THIF. (**A**) The cells were pretreated with 6,7,4'-THIF for 1 h and irradiated with sUV. After 5 min, the cells were lysed, and the phosphorylated PKCα level was evaluated; (**B**) Each active PKCα and PKCδ was co-incubated with 6,7,4'-THIF at the indicated concentrations for 30 min at 30 °C, then MBP, a substrate of PKCα and PKCδ, and [γ-^32^P]-ATP were added to the mixture and additionally incubated for 10 min. The incorporated radiolabeled phosphate was measured using a scintillation counter. Data are represented as the means ± SD, as determined from three independent experiments. Means with different letters (a–c) within a graph were significantly different from each other at *p* < 0.05; (**C**) 6,7,4'-THIF directly binds to endogenous PKCα. The binding of 6,7,4'-THIF with PKCα was visualized using immunoblotting with a specific PKCα antibody: Lane **1** (input control), whole-cell lysates from NHDF; Lane **2** (control), lysates from NHDF; and Lane **3**, whole-cell lysates from NHDF cells precipitated with 6,7,4'-THIF-Sepharose 4B beads; and (**D**) 6,7,4'-THIF does not compete with ATP for binding with PKCα. Active PKCα was incubated with ATP at different concentrations (10, 100 and 1000 µM) with 100 µL of 6,7,4'-THIF-Sepharose 4B beads or 100 µL of Sepharose 4B (as a negative control) overnight. After washing, the binding was confirmed by western blot.

PKC is a well-known upstream regulator of MAPKKs. Recently, several papers have indicated that PKCα mediates MMP-1 expression by activating various signaling pathways [23,27]. Additionally, PKC kinase activity and the protein level of PKCα in human skin fibroblasts from older donors is increased compared to that of younger donors, suggesting its potential association with the skin aging process [27]. Thus, we hypothesized that PKCα is a potential target of 6,7,4'-THIF in the regulation of MMP-1 expression. In Figure 4A, although sUV exposure enhanced phosphorylation of PKCα, 6,7,4'-THIF did not affect sUV-induced PKCα phosphorylation. Next, we evaluated the effect of 6,7,4'-THIF on PKCα kinase activity. Treatment with 6,7,4'-THIF suppressed the kinase activity of PKCα in a dose-dependent manner (Figure 4B), whereas PKCδ kinase activity was not significantly suppressed by 6,7,4'-THIF. To verify if 6,7,4'-THIF directly interacts with PKCα, an* ex vivo* pull-down assay was conducted using 6,7,4'-THIF conjugated with Sepharose 4B in NHDFs cell lysate. Our result showed that 6,7,4'-THIF directly binds with PKCα in cell lysate, and this interaction was not diminished by ATP (Figure 4D). Overall, we demonstrated that 6,7,4'-THIF inhibits sUV-induced MMP-1 expression by directly suppressing PKCα kinase activity.

### 2.4. PKCα Plays a Crucial Role in sUV-Induced MMP-1 Expression in NHDFs

**Figure 5:**
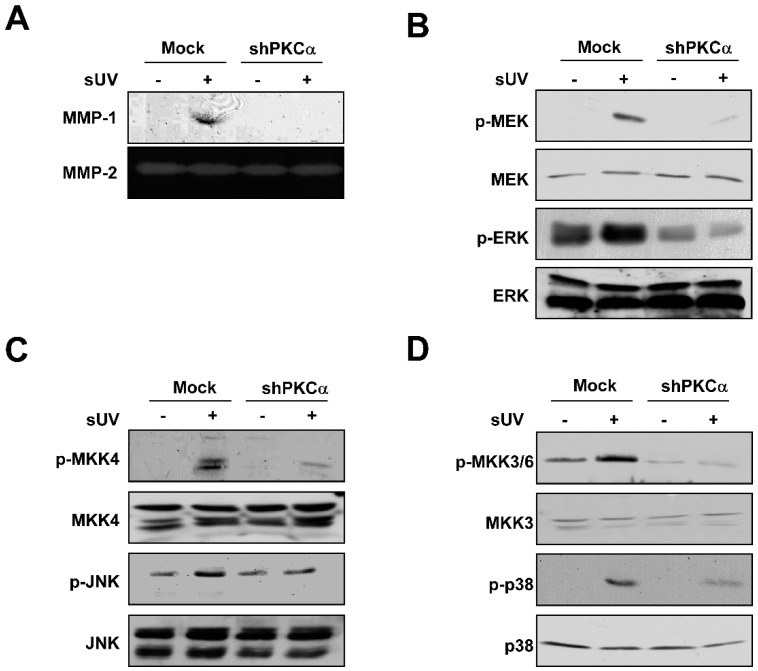
PKCα modulates sUV-induced MMP-1 expression by activation of the MAPK signaling pathway. (**A**) sUV could not induce MMP-1 expression in shPKCα-NHDF cells compared with shMock-NHDF cells. After 48 h of sUV irradiation, the media were collected, and the MMP-1 expression was analyzed using western blot analysis, as described in the Materials and Methods; PKCα regulates the sUV-induced MEK/ERK (**B**), MKK4/JNK (**C**) and MKK3/6/p38 (**D**) signaling pathways. After starvation with serum-free DMEM for 24 h, shMock and shPKCα-NHDF cells were exposed to sUV or not. Then, the protein was harvested after 15 min (p-MEK, p-MKK4 and p-MKK3/6) or 30 min (p-ERK, p-JNK and p-p38). The level of protein was visualized using specific antibodies.

Among PKC isoforms, the increased protein level of PKCα was observed in older donor’s skin fibroblasts [27]. Hence, we determined whether PKCα is closely related in sUV-induced MMP-1 expression in the current model using shPKCα-NHDFs. While the MMP-1 expression level was increased by sUV irradiation in shMock, the exposure of sUV did not induce MMP-1 expression in shPKCα-NHDFs (Figure 5A). To determine whether PKCα regulates the sUV-induced MAPK signaling pathway, we evaluated the phosphorylation levels of MAPKs in shPKCα-NHDFs. Contrary to the results in shMock-NHDFs, the MEK-ERK, MKK4-JNK and MKK3/6-p38 signaling pathways were not activated by sUV irradiation in shPKCα-NHDFs (Figure 5B–D). Taken together, PKCα plays a critical role in sUV-induced MMP-1 expression as an upstream regulator of the MEK-ERK, MKK4-JNK and MKK3/6-p38 signaling pathways in NHDFs.

### 2.5. Discussion

Soybeans are an attractive source for cosmetics due to their health benefits and safety [28]. Various cosmetic products have been developed incorporating soybean ingredients, such as soybean fiber, peptide and isoflavone [29]. As a differentiation strategy in the market, companies use fermentation and bioconversion technology to enhance the effect and value of soybean materials [21]. Since many types of fermented soybean foods have been used for a long time and various methods of fermentation and bioconversion have already been developed, the fermentation and bioconversion of soybeans is relatively easier than other sources. During the fermentation and bioconversion of soybeans, isoflavone, which is a major active component in soybeans, is converted to a variety of derivatives [30]. 6,7,4'-THIF is one of the derivatives converted from daidzein, which is a well-known soy isoflavonoid [21]. Oxidation of daidzein at the six carbon of the A ring generates 6,7,4'-THIF, which is medicated by the 3 cytochrome P450 enzyme, including CYP1A2, CYP1A1 and CYP1B1 [31]. 6,7,4'-THIF could be produced industrially by bioconversion using these enzymes from daidzein. If the anti-aging effects of 6,7,4'-THIF are verified, 6,7,4'-THIF will be a valuable cosmetic material.

**Figure 6:**
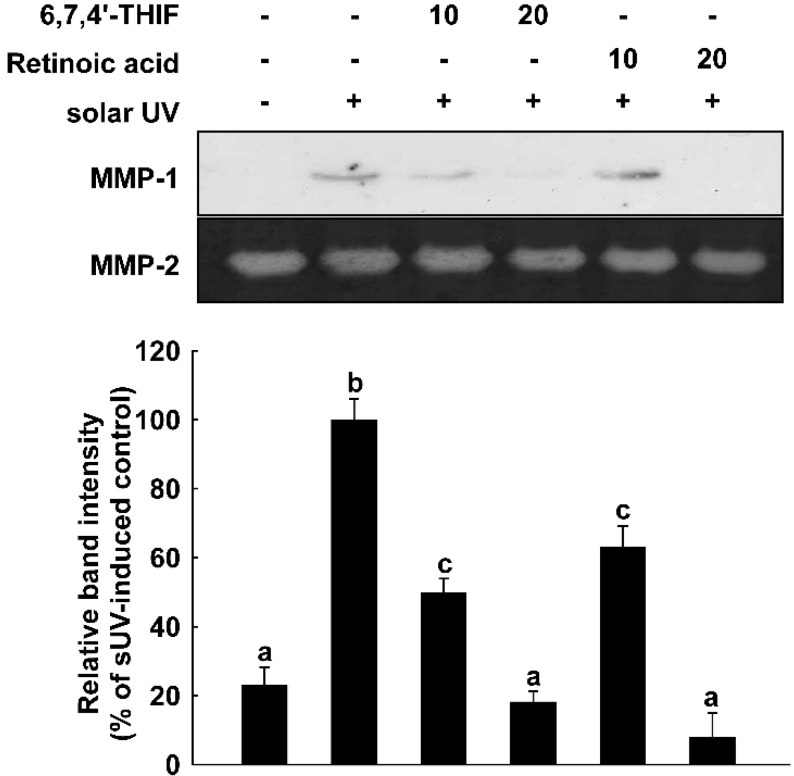
6,7,4'-THIF shows a better inhibitory effect than retinoic acid on sUV-induced MMP-1 expression. The cells were pretreated with 6,7,4'-THIF for 1 h and irradiated with sUV. The media was collected after 48 h of sUV exposure. The MMP-1 expression level was measured by western blot analysis as described in the Materials and Methods. Means with different letters (a–c) within a graph were significantly different from each other at *p* < 0.05.

A previous study reported daidzein’s potential as an anti-skin aging agent [19]. Although daidzein did not show an inhibitory effect on the sUV-induced MMP-1 expression level in our results (Figure 2A), its bioconversion product, 6,7,4'-THIF, significantly suppressed sUV-induced MMP-1 expression. This result is along the same lines with previous literature showing the improved bioactive function of bioconverted soybean products and compounds. Lee* et al.*, have shown that fermented soybean has a stronger inhibitory effect on MAPKs than raw soybean [32]. Additionally, 6,7,4'-THIF revealed a better inhibitory effect on MMP-1 expression than retinoic acid, an industrial anti-wrinkle ingredient (Figure 6). This notion indicated that 6,7,4'-THIF can be a new anti-skin aging compound. We previously showed that another daidzein metabolite, 7,3',4'-THIF, is more effective at inhibiting Ultravioloet B (UVB)-induced cyclooxygenase-2 (COX-2) expression than daidzein [16].

We suggested that PKCα is a direct molecular target of 6,7,4'-THIF on sUV-induced MMP-1 expression. Direct inhibition of PKCα by 6,7,4'-THIF reduced sUV-induced MAPK signaling and MMP-1 expression. PKCα, a member of the serine/threonine kinase PKC family, has been implicated in various biological activities, such as cell survival and proliferation [33,34,35,36,37,38,39,40]. Interestingly, PKC activity is increased in fibroblasts with age. Total PKC activity was shown to be four-times higher in human skin fibroblasts of 61-year-old donors than those of one-week-old donors [27]. This study also showed that only the PKCα protein level is increased with age using specific antibodies against PKC isoforms (α, δ, ε and ζ). In accordance with these results, the protein level of PKCα is up-regulated in aged* in vitro* cell.

PKCα is expressed in human keratinocytes, and the inhibition of PKC using pharmacological inhibitors suppressed heat-induced MMP-1 expression [23]. The decreased MMP-1 expression was shown in siPKCα-HaCaT, but not in siPKCβ- or siPKCδ-HaCaT cells. The parallel expression level of MMP-1 with the PKCα protein level was also reported. Indeed, our data showed that sUV could not induce MMP-1 expression in shPKCα-NHDF. In addition, the MEK/ERK, MKK4/JNK and MKK3/6/p38 signaling pathways were not activated by UV in shPKCα-NHDF cells. Overall, we confirmed that PKCα plays a critical role in MMP-1 expression by sUV irradiation by activating the MEK/ERK, MKK4/JNK and MKK3/6/p38 signaling pathways. Furthermore, we revealed that PKCα is the direct target of 6,7,4'-THIF by demonstrating the inhibition of PKCα kinase activity by 6,7,4'-THIF and the direct interaction of 6,7,4'-THIF with PKCα in cell lysate. We also elucidated that 6,7,4'-THIF binds to the non-ATP binding site of PKCα. The result of ATP-independent binding of 6,7,4'-THIF to PKCα indicates that 6,7,4'-THIF can inhibit PKCα kinase activity in an allosteric manner. However, to further verify how 6,7,4'-THIF interacts with PKCα, a structural study is required utilizing X-ray crystallography and computer modeling. Additionally, although our data support the biological activity and mechanisms of 6,7,4'-THIF* in vitro*,* in vivo* and clinical studies are needed to further validate its anti-skin aging effect.

Overall, we demonstrated that 6,7,4'-THIF may exert an anti-photoaging effect by suppressing MMP-1, which plays a key role in wrinkle formation. In addition, we revealed that the inhibitory effect of 6,7,4'-THIF on MMP-1 results from its direct interaction with and suppression of PKCα.

## 3. Materials and Methods

### 3.1. Materials

6,7,4'-THIF was obtained from Chromadex™ (Irvine, CA, USA), and Dulbecco’s modified eagle medium (DMEM) and MMP-1 antibody were purchased from Thermo Fisher Scientific (San José, CA, USA). Medium 199 (M199) and daidzein were purchased from Sigma-Aldrich (St. Louis, MO, USA), and fetal bovine serum (FBS) was purchased from Gemini Bio-Products (Calabasas, CA, USA). CNBr-Sepharose 4B, [γ-32P]-ATP and the chemiluminescence detection kit were obtained from Amersham Pharmacia Biotech (Piscataway, NJ, USA). The protein assay kit was purchased from Bio-Rad Laboratories (Hercules, CA, USA). MTS solution was from Promega (Madison, WI, USA). Penicillin/streptomycin was purchased from Invitrogen (Grand Island, NY, USA). Primary antibodies recognizing phosphorylated MEK (Ser^217/221^), total MEK, phosphorylated SEK1/MKK4 (MKK4, Ser^257^/Thr^261^), phosphorylated MKK3 (Ser^189^)/6 (Ser^207^), total MKK3, phosphorylated p38 (Tyr^180/182^), total p38 and PKCα were purchased from Cell Signaling Technology (Danvers, MA). Antibodies against phosphorylated-ERKs (Tyr^204^), total ERKs, ERK2, total MKK4, phosphorylated JNK (Thr^183^/Tyr^185^), total JNK, JNK2 and phosphorylated PKCα (Ser^657^) were obtained from Santa Cruz Biotechnology (Santa Cruz, CA, USA). Active PKCα and PKCδ proteins were purchased from Millipore (Bedford, MA, USA).

### 3.2. Cell Culture

Normal human dermal fibroblasts (NHDF) cells were obtained from the American Type Culture Collection (Manassas, VA, USA). The cells were cultured in DMEM/M199 (4:1) supplemented with 10% (*v*/*v*) FBS under 37 °C, 5% CO_2_ conditions.

### 3.3. Cell Cytotoxicity

To examine the cytotoxicity of daidzein and 6,7,4-THIF on NHDF cells, the cells were seeded in 96-well plates. Upon reaching confluency, the cells were starved with serum-free DMEM for 24 h. After incubation with the various doses of daidzein or 6,7,4-THIF for 24 h, the cytotoxicity of the compounds was analyzed with Cell Titer 96 Aqueous One Solution (Promega). In brief, the cells were incubated with 20 mL of MTS solution for 1 h at 37 °C in a 5% CO_2_ incubator. The absorbance was evaluated at 492 nm.

### 3.4. Solar UV Irradiation

The solar UV resources are UVA-340 lamps (Q-Lab Corporation; Cleveland, OH, USA). The region of the wavelength is from 295 to 365 nm with the major peak emission of 340 nm. The percentage of UVA and UVB of the sUV source was measured using a UV meter as 94.5% and 5.5%, respectively. The dose of sUV used in the current study was 60 kJ/m^2^. For sUV irradiation, the media were changed with serum-starved DMEM/M199 (4:1) before sUV exposure. Then, the plate cover was taken off for direct sUV irradiation of the cells.

### 3.5. Western Blot Analysis

NHDF cells were cultured to confluence and starved with serum-free DMEM for 24 h. Additionally, the 6,7,4'-THIF was added to NHDF at various doses (10 and 20 µM) for 1 h. The cells were exposed to sUV, subsequently. Equal amounts of proteins were separated on 10% SDS-polyacrylamide gels. Additionally, the proteins were transferred to Immobilon P membranes (Millipore). The membranes were blocked with 5% fat-free milk for 1 h and incubated with specific primary antibodies at 4 °C overnight. Next, the proteins were hybridized with HRP-conjugated secondary antibody, and the light emission was detected using a chemiluminescence detection kit (GE Healthcare, Pittsburgh, PA, USA).

### 3.6. Lentiviral Infection

To generate the knocked down PKCα of NHDF, a shRNA system was used. The lentiviral expression vectors, including Gipz-shPKCα (RNAi core, University of Minnesota, Minneapolis, MN, USA), and packaging vectors, including pMD2.0G and psPAX, were purchased from Addgene Inc. (Cambridge, MA, USA). To prepare shPKCα viral particles, each viral vector and the packaging vectors (pMD2.0G and psPAX) were transfected into HEK293T cells using jetPEI following the manufacturer’s instructions. The transfection medium was changed at 4 h after transfection, and the cells were then cultured for 36 h. The viral particles were harvested by filtration using a 0.45-mm syringe filter, then combined with 8 μg/mL polybrene (EMD Millipore) and infected into 60% confluent NHDF cells overnight. The cell culture medium was replaced with fresh complete growth medium for 24 h before the cells were selected for using puromycin (Sigma, St Louis, MO, USA, 2 μg/mL) over 36 h. The selected cells were then used for further experiments.

### 3.7. Zymography

The activity of MMP-2 was evaluated using zymography. Zymography was performed using 10% polyacrylamide gels in the presence of gelatin (0.5 mg/mL) as a substrate for MMP-2. The samples were suspended in loading buffer (10% SDS, 25% glycerol, 0.25 M Tris (pH 6.8) and 0.1% bromophenol blue) and loaded onto 10% SDS-PAGE gels without denaturation. After electrophoresis, the gels were incubated in renaturing buffer (Invitrogen) at room temperature for 30 min and then incubated for 24 h at 37 °C in developing buffer (Invitrogen). The gels were then stained with 0.5% Coomassie Brilliant Blue.

### 3.8. In Vitro Kinase Assay

PKCα and δ kinase assays were performed using active recombinant PKCα and δ enzymes following the manufacturer’s instructions. Briefly, active PKCα or δ and 6,7,4'-THIF were incubated at 30 °C for 15 min in assay buffer (20 mM MOPS (pH 7.2), 25 mM β-glycerol phosphate, 5 mM EGTA, 1 mM sodium orthovanadate and 1 mM DTT). Two millimeters of myelin basic protein (MBP) substrate were added to each mixture, then incubated at 30 °C for 15 min with [γ-32P]-ATP solution in a magnesium acetate-ATP cocktail buffer (Upstate Biotechnology Inc., Lake Placid, NY, USA). Next, the reaction mixture was transferred onto p81 paper. Using 0.75% phosphoric acid, the p81 paper was washed three times for 5 min. The radio-labeled phosphate was detected using a scintillation counter.

### 3.9. 6,7,4'-THIF Pull-Down Assay Using Sepharose 4B

6,7,4'-THIF-Sepharose 4B complex was generated by following the procedure described in the previous literature [41]. The NHDF cell lysate was incubated with this 6,7,4'-THIF-Sepharose 4B complex overnight at 4 °C. The binding of the protein to 6,7,4'-THIF-Sepharose 4B was examined by western blot analysis.

### 3.10. ATP and PKCα Competition Assay

Recombinant PKCα (200 ng) was incubated with ATP (10, 100 and 1000 µM) at 4 °C for 1 h. Sepharose 4B (negative control) or 6,7,4'-THIF-Sepharose 4B was added to the mixture and incubated 4 °C overnight. Extra proteins were washed with wash buffer, and PKCα was detected by western blot analysis.

## 3.11. Statistical Analysis

Data were expressed as the means ± standard deviation (SD). One-way analysis of variance (ANOVA) with Tukey’s HSD test was used to evaluate mean differences of group and statistical significance. Differences were considered significant at *p* < 0.05.
